# EFFECTS OF A STAGED INTEGRAL ART-BASED COGNITIVE INTERVENTION FOR OLDER ADULTS ON THE ALZHEIMER’S DISEASE SPECTRUM

**DOI:** 10.1093/geroni/igae098.3616

**Published:** 2024-12-31

**Authors:** Yuanjiao Yan, Chenshan Huang, Yanhong Shi, Rong Lin, Hong Li

**Affiliations:** Fujian Provincial Hospital, Fuzhou, Fujian, China (People’s Republic); Fujian Medical University, Fuzhou, Fujian, China (People’s Republic); Fujian Medical University, Fuzhou, Fujian, China (People’s Republic); Fujian Medical University, Fuzhou, Fujian, China (People’s Republic); Fujian Medical University, Fuzhou, Fujian, China (People’s Republic)

## Abstract

**Background:**

The combined art activities can prevent or reverse the cognitive decline. However, few studies have systematically examined its effects. To evaluate the effects of a staged integral art-based cognitive intervention (SIACI) in older adults on the Alzheimer’s disease (AD) spectrum.

**Methods:**

Participants were randomised in a 1:1 ratio into an intervention group (n=72) and a wait-list control group (n=72). The intervention group underwent a 16-week, 24-session SIACI program, while the control group underwent the program after the follow-up assessment. General and specific domains of cognitive function, and other health-related outcomes were measured at baseline (T0), immediately after the intervention (T1), and 6 months follow-up (T2).

**Results:**

Of the 144 participants (mean [SD] age, 71.6 [5.8] years; 50 [34.7%] males and 94 [65.3%] females), 130 and 115 completed the questionnaires at T1 and T2, respectively. The average attendance rate in the intervention group was 86.0%. At T1, the intervention group showed greater improvement than the control group in general cognitive functions (MoCA, between-group mean difference, 95% CIs, 0.4 to 2.5, p=0.009). Additionally, the intervention group demonstrated statistically significant improvement compared to the control group in language, memory, quality of life, sleep quality and physical activity level at T1. Statistically significant group differences remained in sleep quality (PSQI, between-group mean difference, -1.3 [95% CIs, -2.5 to -0.1], p=0.035) at the 6-month follow-up. Conclusions: In this randomised controlled trial of the SIACI program, older adults on the AD spectrum who participated in the program experienced improved cognition and psychological health.

